# Reluctant reviewers? Publons may help

**DOI:** 10.1002/nop2.143

**Published:** 2018-03-25

**Authors:** Roger Watson

*Nursing Open* continues to grow, submissions are frequent and regular, and we are putting ever larger issues online. Naturally, the volume of submissions to *Nursing Open* does not match those to my other journal *JAN*, but the latter is long established, has a relatively high impact factor and an international reputation. *Nursing Open* is still new and along with growing the volume of submissions and the size of our issues, reputation takes longer. Another difference I find between *Nursing Open* and *JAN* is that finding reviewers for manuscripts is much easier for *JAN*. My *JAN* team say that it takes six to eight attempts to find reviewers for *JAN* manuscripts. At *Nursing Open* it takes many more and this can contribute to delays in returning reviews to authors. If you are an author who has, or is, experiencing a delay in waiting for a decision on your manuscript then I not only apologize but also want to assure you that the team of associate editors is aware and we do all we can to recruit new reviewers, find suitable reviewers from our pool and to follow‐up with reviewers where they are slow and to replace reviewers who have declined to review.

Perhaps you are a reluctant reviewer, you may even have declined to review manuscripts for *Nursing Open*. If so, I would like to draw to your attention a service that ensures you receive credit for your reviews in a way that is recordable and presentable, for example, at appraisals by academic managers. Publons makes visible your activities as a reviewer, and Wiley—who publish *Nursing Open* are partners with Publons and fully committed to what it offers. According to the webpage:

“Publons helps you get the recognition you deserve for keeping watch over science and research. Easily import, verify, and store a record of every peer review you perform and every manuscript you handle as an editor, for any journal in the world, in full compliance with all editorial policies.”

So, if you have been reluctant to review for Nursing Open, or for any of the journals from publishers partnered to Publons then I would encourage you to visit the Publons webpage and register, Thereafter, you will be prompted, during the review process of partnered journals to record your review with Publons or this step can be automated. What results is a personalized webpage to which you can provide a link on your CV or in your employer appraisal documents to demonstrate your important contribution to your scholarly community. And, just in case you were wondering, it is the fact that you performed the review for a specific journal that is recorded—no other details such as the title of the manuscript or the recommendations or comments you made are recorded.

